# Self-reported Morisky Eight-item Medication Adherence Scale for Statins Concords with the Pill Count Method and Correlates with Serum Lipid Profile Parameters and Serum HMGCoA Reductase Levels

**DOI:** 10.7759/cureus.6542

**Published:** 2020-01-02

**Authors:** Abhinav Grover, Mansi Oberoi, Harmeet Singh Rehan, Lalit K Gupta, Madhur Yadav

**Affiliations:** 1 Internal Medicine, University of California, Irvine School of Medicine, Irvine, USA; 2 Internal Medicine, University of South Dakota, Sanford School of Medicine, Sioux Falls, USA; 3 Pharmacology, Lady Hardinge Medical College, New Delhi, IND; 4 Medicine, Lady Hardinge Medical College, New Delhi, IND

**Keywords:** atorvastatin, compliance, dyslipidemia, hmgcoa reductase, rosuvastatin, morisky medication adherence scale

## Abstract

Background

It is imperative that non-compliance with statins be identified and addressed to maximize their clinical benefits. Patient self-reporting methods are convenient to apply in clinical practice but need to be validated.

Objective

We studied the concordance of a patient self-report method, Morisky eight-item medication adherence scale (MMAS)), with the pill count method in measuring adherence with statins and their correlation with extended lipid profile parameters and serum hydroxyl-methylglutaryl coenzyme A reductase (HMGCoA-R) enzyme levels.

Methods

MMAS and the pill count method were used to measure the adherence with statins in patients on statins for any duration. Patients were subjected to an estimation of extended lipid profile and serum HMGCoA-R levels at the end of three months follow-up.

Results

Out of a total of 200 patients included in the study, 117 patients had a low adherence (score less than 6 on MMAS) whereas 65 and 18 patients had medium (score 6 or 7) and high adherence (score of 8), respectively. The majority of patients who had low adherence to statins by MMAS were nonadherent by the pill count method yielding a concordance of 96.5%. Medium or high adherence to statins by the MMAS method had a concordance of 89.1% with the pill count method. The levels of total cholesterol, low-density lipoprotein-cholesterol, apolipoprotein B, and HMGCoA-R were negatively correlated with compliance measured by pill count and MMAS in a statistically significant way and with similar correlation coefficients. HMGCoA-R levels demonstrated a plateau phenomenon, with levels being 9-10 ng/ml when compliance with statin therapy was greater than 60% by pill count and greater than 6 on the Morisky scale.

Conclusion

In conclusion, MMAS and the pill count method showed concordance in measuring adherence to statins. These methods need to be explored further for their interchangeability as surrogates for biomarker levels.

## Introduction

Statins are one of the most effective lipid-lowering agents, which reduce the risk of coronary events by 17%-26% [1]. The benefits of statins are lost when patients are poorly compliant, and it is reported that only 50% of patients who are being treated with statins continue to use their medication at six months, and only 30% to 40% do so after one year [2-3]. Hence, it is imperative that non-compliance with statins be identified to optimize the clinical benefit of statins. Medication reconciliation is often limited by compliance [4]. Medication adherence can be assessed by pill count, pharmacy fill rates, surrogate marker levels, patient self-reporting methods, e.g. Morisky medication adherence scale eight-item (MMAS) and so on. [5]. The most convenient ones are patient self-reporting methods, which can be easily applied in the clinical setting but they are generally not specific for a disease or drug [6-7]. There is a need to understand and validate MMAS for assessing compliance with statins in dyslipidemic patients. Hence, the aim of the present study was to evaluate the concordance of MMAS with the pill count method in measuring compliance with statins in dyslipidemic patients and to assess the correlation of compliance with statins with lipid profile and serum 3-hydroxyl-3-methylglutaryl coenzyme A reductase (HMGCoA-R) enzyme levels [8].

## Materials and methods

Subjects

Dyslipidemic patients with age greater than or equal to 18 years, elevated low-density lipoprotein (LDL) levels and/or triglyceride (TG) levels, and/or low high-density lipoprotein (HDL) levels as per American College of Cardiology/American Heart Association (ACC/AHA) guidelines (2) and on statin therapy for any duration were included in the study. Patients who had acute coronary syndrome (ACS) within the last three months, a history of hypothyroidism, pregnancy/lactation, and hypersensitivity or intolerance to statins were excluded from the study.

Study design

**Figure 1:**
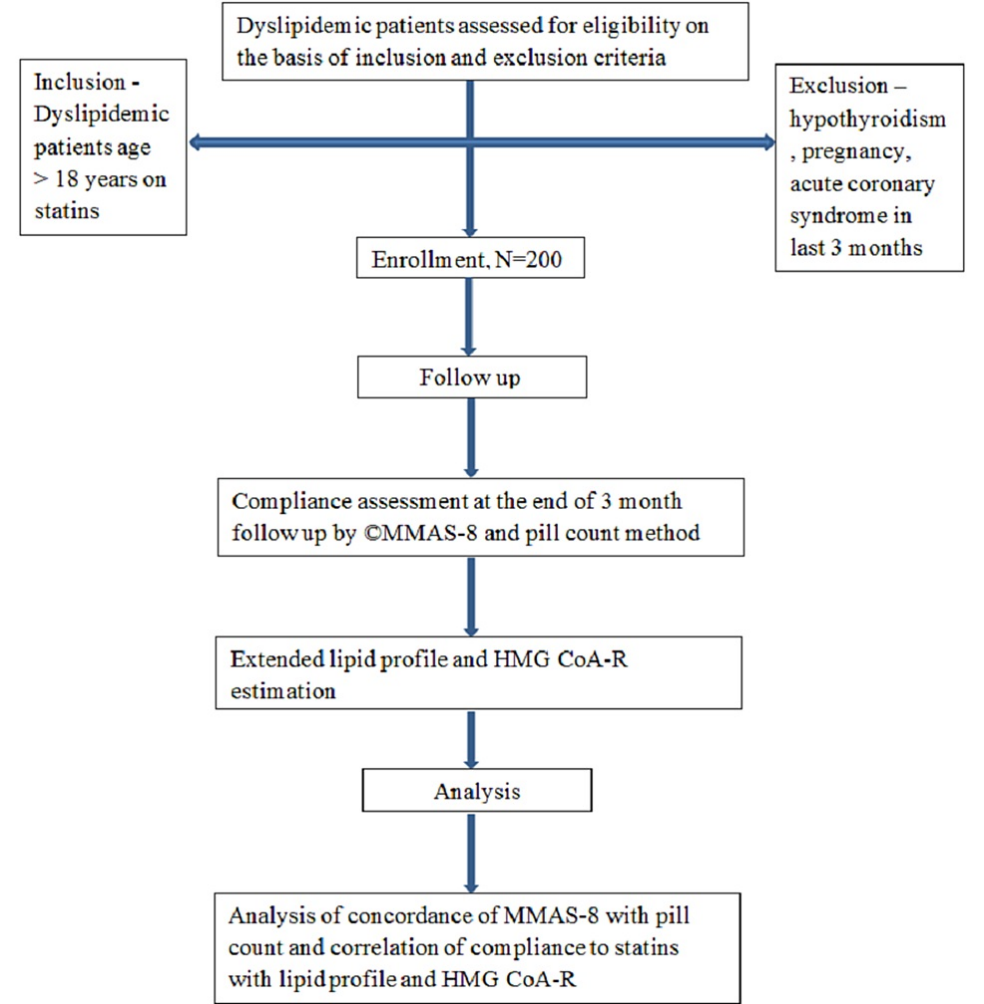
Study design HMG CoA-R = 3-hydroxyl-3-methylglutaryl coenzyme A reductase; MMAS = Morisky medication adherence scale

In a prospective observational fashion, information on patients’ personal, demographic, and socioeconomic status was recorded. They were assessed for compliance with statins using MMAS and the pill count method at the end of three months after refilling medicine [9]. Patients with a score of pill count ≥60% were considered compliant, whereas based on MMAS scores, the patients were categorized into three levels of adherence, i.e. high adherence (score 8), medium adherence (score 6 or 7), and low adherence (score <6) to facilitate its use (Figure 1) [8].

The study protocol was approved by the Institutional Human Ethical Committee. Written informed consent was taken from all the patients. The decision to start the statins or to escalate their doses if required was at the discretion of the treating physician. Patients who required dose modification from one intensity of statin therapy to another within the follow-up period were excluded from the study.

A 5 ml venous blood sample of all the patients was taken to analyze extended lipid profile parameters, including total cholesterol (TC), LDL, TG, HDL, apolipoprotein A1, apolipoprotein B, and serum HMG CoA-R enzyme levels using the enzyme-linked immunosorbent assay (ELISA) method with the BMAssay (Beijing, China) HMGCoA-R kit and the AssayPro (St Charles, Missouri) apolipoprotein A1 (ApoA1) and apolipoprotein B (ApoB) ELISA kits.

Statistical considerations

The compliance data are presented as percentages for pill count and as mean of proportions for MMAS, whereas lipid profile parameters were presented as mean±standard deviation and the agreement between MMAS and pill count was described by percentage concordance. The trend of pill count across MMAS categories was analyzed using the least square and maximum likelihood ratio for discrete and continuous variables, respectively. The Pearson’s correlation analysis was used for correlation of compliance with lipid profile and serum HMG CoA reductase levels. A p-value of <0.05 was considered statistically significant.

## Results

Out of the total of 200 patients included in the study, 101 (50.5%) were females. The overall mean age of all the patients was 55.15±10.23 years (range, 23 to 82 years). The mean duration of prescription for statin at the time of enrollment was 8.6±13.08 months (range, 1-72 months). The demographic and clinical characteristics of patients by the low, medium, and high adherence MMAS category are described in Table 1.

**Table 1:** Patients’ demographic and clinical characteristics as per the MMAS category MMAS = Morisky medication adherence scale; S.D. = standard deviation

	MMAS category (score range)			p-value
	Low (<6) (N=117)	Medium (6 to <8) (N=65)	High (8) (N=18)	
Age (years) (Mean ± S.D)	54.7 ± 9.9	55.9 ± 11.1	55.3 ± 11.5	0.82
Gender females, N (%)	59 (50.4)	32 (49.2)	10 (55.5)	0.86
Comorbid conditions				
Diabetes mellitus, N (%)	76 (64.9)	46 (70.7)	14 (77.7)	0.50
Hypertension, N (%)	60 (51.2)	31 (47.7)	4 (22.2)	0.07
Ischemic heart disease, N (%)	11 (9.4)	4 (6.2)	1 (5.5)	0.71

Low adherence to statins by the MMAS method was observed in 117 patients who also showed no adherence by the pill count method (n=113) yielding concordance of 96.5%. There were 83 patients who had medium or high adherence to statins by the MMAS method and 74 of these were adherent by the pill count method yielding concordance of 89.1%. When the low and medium adherence categories by MMAS were clubbed together, their concordance with nonadherence by pill count was 67%, whereas the concordance of high adherence by MMAS and adherence by pill count was 100% (Table 2).

**Table 2:** Comparison of statin compliance by MMAS and the pill count method MMAS = Morisky medication adherence scale; S.D. = standard deviation

Parameters	Pill count method	MMAS category ( score range)			p-value
		Low (<6)	Medium (6 to <8)	High (8)	
Number of patients (N)	200	117	65	18	
Mean pill count (mean±S.D.)	56.71	45.10±10.69	68.42±9.46	89.87±3.16	0.000
Number of non-adherers (pill count <60%) (N)	121	113/117 (96.58%)	9/65 (13.84%)	0/18 (0%)	0.000
Number of adherers (pill count≥60%) (N)	79	4/117 (3.41%)	56/65 (86.15%)	18/18 (100%)	0.000

The levels of total cholesterol, LDL, HMG CoA-R, and Apo B were negatively correlated with compliance measured by pill count and MMAS in a statistically significant manner. The levels of HDL-C were positively correlated with compliance by both measures. Serum LDL levels show similar negative correlation with compliance by MMAS (r=-0.750, p=0.000) and pill count (r=-0.776, p=0.000). HMG CoA-R levels also show similar negative correlation with compliance by MMAS (r=-0.497, p=0.000) and pill count (r=-0511, p=0.000). And Apo B shows negative correlation with MMAS (r=-0.239, p=0.001) and pill count (-0.233, p=0.001) (Table 3).

**Table 3:** Correlation of compliance by MMAS-8 and the pill count method with extended lipid profile and HMGCoA-R MMAS = Morisky medication adherence scale; TC = total cholesterol; TG = triglyceride; LDL = low-density lipoprotein; HDL = high-density lipoprotein; HMGCoA-R = 3-hydroxyl-3-methylglutaryl coenzyme A reductase; ApoA1 = Apolipoprotein A1; ApoB = Apolipoprotein B

Method to study adherence		TC	TG	LDL	HDL	HMGCoA-R	ApoA1	ApoB
MMAS-8	Pearson Correlation	-0.643	-0.487	-0.750	0.781	-0.497	-.025	-0.239
	Sig. (2-tailed)	0.000	0.000	0.000	0.000	0.000	0.727	0.001
Pill count	Pearson Correlation	-0.602	-0.498	-0.776	0.785	-0.511	-0.039	-0.233
	Sig. (2-tailed)	0.000	0.000	0.000	0.000	0.000	0.583	0.001

**Figure 2:**
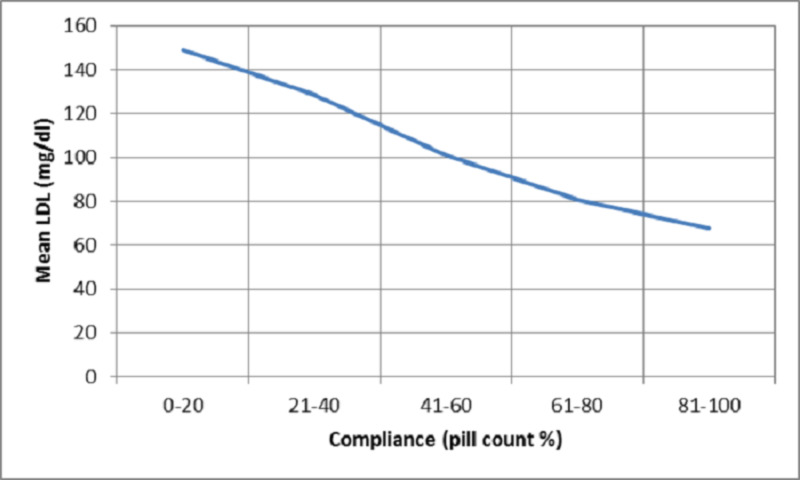
Plot of mean serum LDL levels against compliance measured using pill count LDL = low-density lipoprotein

**Figure 3:**
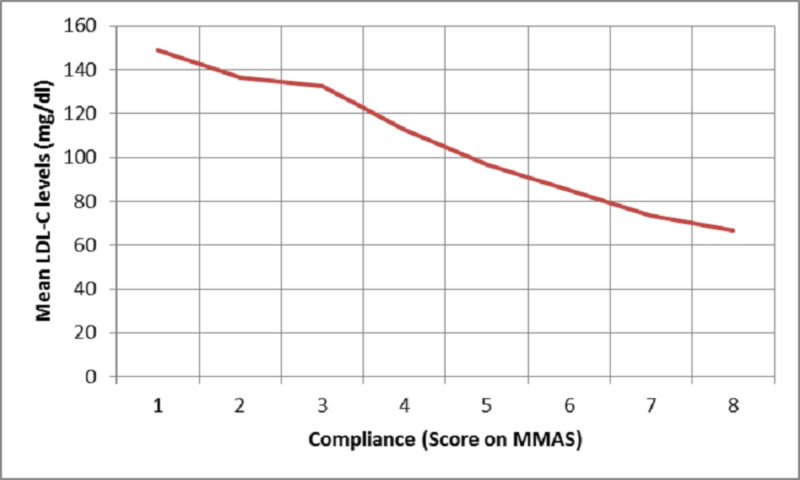
Plot of mean serum LDL levels against compliance measured using MMAS LDL = low-density lipoprotein; MMAS = Morisky medication adherence scale

**Figure 4:**
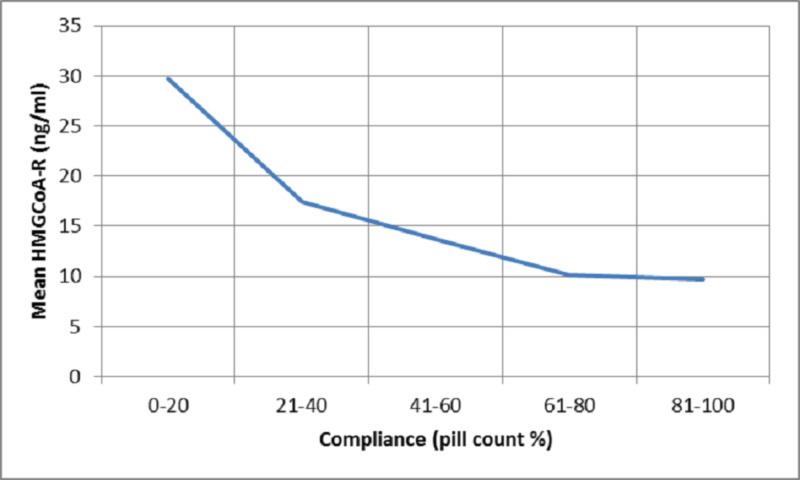
Plot of mean serum HMG CoA-R levels against compliance measured using pill count HMG CoA-R = 3-hydroxyl-3-methylglutaryl coenzyme A reductase

**Figure 5:**
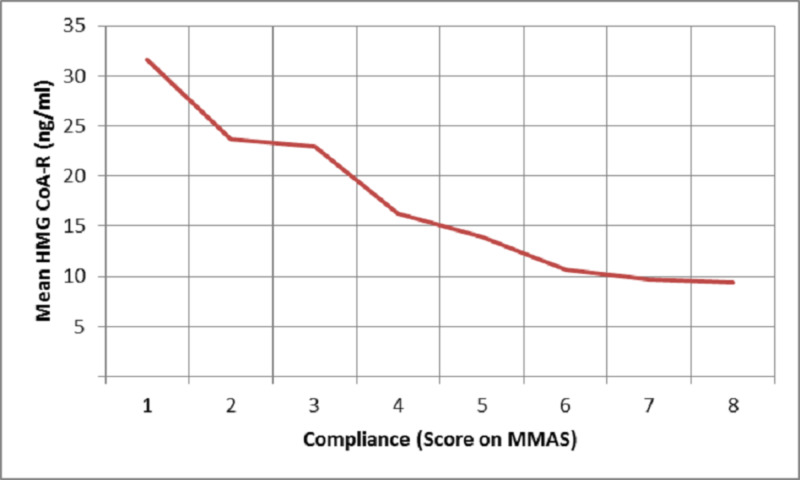
Plot of mean serum HMG CoA-R levels against compliance measured using MMAS HMG CoA-R = 3-hydroxyl-3-methylglutaryl coenzyme A reductase; MMAS = Morisky medication adherence scale

HMG CoA-R levels demonstrated a plateau phenomenon with levels being 9-10 ng/ml when compliance with statin therapy was greater than 60% by pill count and greater than 6 on the Morisky scale whereas the LDL-C levels achieved were 60-80 mg/dl with increase in compliance beyond these levels (Figures 2-5).

## Discussion

Prevalence of non-compliance with statins

Compliance with statins has been reported to be around 50% at the end of one year of initiation of treatment [10]. Clinicians require information on medication adherence to draw proper conclusions about the effectiveness of treatment. The goal is to have access to a quick, reasonably accurate self-report adherence measure for use in outpatient settings to facilitate clinical decision-making. A quick and accurate assessment of compliance can be especially beneficial for dyslipidemic patients in choosing between measures to increase compliance or switching to a higher intensity of statins [11].

Compliance assessment for statins using MMAS and the pill count method

We evaluated the association and concordance of MMAS with pill count in measuring compliance with statins, as no other study is available that evaluated this aspect. The majority of patients who had low adherence to statins by MMAS were nonadherent by the pill count method showing concordance whereas medium or high adherence to statins by MMAS yielded a concordance of 89.1% (Table 2). When the low and medium adherence categories by MMAS were clubbed together, their concordance with non-adherence by pill count was 67% whereas the concordance of high adherence by MMAS with adherence by pill count was 100% (Table 2). This suggested a score of lower than 6 on MMAS is a better predictor of non-adherence than defining non-adherence as having a score of less than 8 on MMAS.

The identification of patients with low adherence may facilitate the implementation of suitable interventions and aid in the optimization of therapeutic benefit. There are numerous behaviors related to non-adherence, which may be modifiable, including lack of mindfulness, forgetting, the complexity of the treatment regimen, and several others [12-20]. Some of these can be identified using the responses of patients to individual items of MMAS and can enable appropriate interventions to improve adherence but this aspect needs to be explored further in dyslipidemic patients [21].

Compliance with statins and correlation with biomarkers of dyslipidemia

We also correlated the compliance with statins derived by MMAS and the pill count method with lipid profile and serum HMG CoA reductase enzyme levels. There was a significant negative correlation between total cholesterol, LDL, HMG CoA-R, and Apo B levels and compliance by pill count and MMAS signifying the comparability of the two methods of compliance in estimating the lipid profile parameters. Whereas, HDL shows a significant positive correlation with compliance by both methods. Serum LDL levels show similar negative correlation with compliance by MMAS (r=-0.750, p=0.000) and pill count (r=-0.776, p=0.000). And Apo B also shows a similar negative correlation with MMAS (r=-0.239, p=0.001) and pill count (-0.233, p=0.001). No study is available to compare the findings of our study. The similar correlation coefficients of different parameters with compliance by MMAS and pill count suggest a parallel in MMAS and the pill count method as measures of compliance.

HMG CoA-R shows a similar negative correlation with compliance by MMAS (r=-0.497, p=0.000) and pill count (r=-0511, p=0.000). Also, the levels of HMG CoA-R show a plateau at levels of 9-10 ng/ml when compliance is beyond 60% by the pill count method and greater than a score of 6 on MMAS (Figures 4-5). This suggests that high adherence defined by either of the two methods correlates with an HMG CoA-R level of 9-10 ng/ml and this cut-off could potentially be explored as a surrogate marker for defining adherence and non-adherence. Also, it would be beneficial in estimating the effectiveness of statins. This warrants the study of the utility of these self-reported methods of compliance to optimize treatment with other dyslipidemia drugs such as ezetimibe and proprotein convertase subtilisin/kexin type 9 (PCSK-9) inhibitors.

Study limitations

There are several limitations of self-reported measures of adherence, including reliance on recall and social desirability bias, with a tendency to overestimate adherence [12]. Pill counts may not be an accurate estimate either since they fail to measure whether their medication was taken on schedule [9]. In our study, hypertension seemed to have a non-statistically significant association, with low levels of compliance possibly due to the multiple medications generally prescribed to these patients.

## Conclusions

MMAS and the pill count method of estimating compliance showed concordance. The compliance assessed by both the methods correlated in a similar manner with the modification in the extended lipid profile levels and with the inhibition of HMG CoA-R levels. In settings where lipid profile and HMG COA-R estimation is not available, if the compliance of statins by MMAS is more than a score of 6 or more than 60%-80% by the pill count method, it can be assumed that the benefit of statins has been adequately extended to the patient.
