# Correction: Self-reported Morisky Eight-item Medication Adherence Scale for Statins Concords with the Pill Count Method and Correlates with Serum Lipid Profile Parameters and Serum HMGCoA Reductase Levels

**DOI:** 10.7759/cureus.c118

**Published:** 2023-05-18

**Authors:** Abhinav Grover, Mansi Oberoi, Harmeet Singh Rehan, Lalit K Gupta, Madhur Yadav

**Affiliations:** 1 Internal Medicine, University of California, Irvine School of Medicine, Irvine, USA; 2 Internal Medicine, University of South Dakota, Sanford School of Medicine, Sioux Falls, USA; 3 Pharmacology, Lady Hardinge Medical College, New Delhi, IND; 4 Medicine, Lady Hardinge Medical College, New Delhi, IND

This article has been corrected at the request of the authors due to an error that appears in Table 2 as well as the Materials and Methods: Study Design section. In both instances, the threshold for defining compliance is incorrectly specified as 80% instead of the correct threshold of 60%. It is mentioned as 60-80% or 60% in both the Results and Conclusion sections, but should have been accurately depicted in Table 2 and the Materials and Methods section as well. The authors and the journal regret that this error was not identified and addressed prior to publication.

